# Computational Analysis of Alternative Photosynthetic Electron Flows Linked With Oxidative Stress

**DOI:** 10.3389/fpls.2021.750580

**Published:** 2021-10-22

**Authors:** Nima P. Saadat, Tim Nies, Marvin van Aalst, Brandon Hank, Büsra Demirtas, Oliver Ebenhöh, Anna Matuszyńska

**Affiliations:** ^1^Institute of Quantitative and Theoretical Biology, Heinrich Heine University Düsseldorf, Düsseldorf, Germany; ^2^CEPLAS - Cluster of Excellence on Plant Sciences, Heinrich Heine University Düsseldorf, Düsseldorf, Germany

**Keywords:** reactive oxygen species, cyclic electron flow, mathematical model, photosynthesis, electron transport (photosynthetic)

## Abstract

During photosynthesis, organisms respond to their energy demand and ensure the supply of energy and redox equivalents that sustain metabolism. Hence, the photosynthetic apparatus can, and in fact should, be treated as an integrated supply-demand system. Any imbalance in the energy produced and consumed can lead to adverse reactions, such as the production of reactive oxygen species (ROS). Reaction centres of both photosystems are known sites of ROS production. Here, we investigate in particular the central role of Photosystem I (PSI) in this tightly regulated system. Using a computational approach we have expanded a previously published mechanistic model of C3 photosynthesis by including ROS producing and scavenging reactions around PSI. These include two water to water reactions mediated by Plastid terminal oxidase (PTOX) and Mehler and the ascorbate-glutathione (ASC-GSH) cycle, as a main non-enzymatic antioxidant. We have used this model to predict flux distributions through alternative electron pathways under various environmental stress conditions by systematically varying light intensity and enzymatic activity of key reactions. In particular, we studied the link between ROS formation and activation of pathways around PSI as potential scavenging mechanisms. This work shines light on the role of alternative electron pathways in photosynthetic acclimation and investigates the effect of environmental perturbations on PSI activity in the context of metabolic productivity.

## 1. Introduction

Photosynthetic organisms are the primary producers of biomass available in the biosphere. By employing complex biophysical processes, which act on multiple temporal and spatial scales, they perform highly efficient energy converting reactions (see for example Ksenzhek and Volkov, 1998). The basic machinery behind these reactions consists of two parts. The first one is the photosynthetic electron transport chain (PETC). Embedded in the thylakoid membrane, the PETC mediates the transfer of electrons, extracted from water molecules, over the complexes of Photosystem II (PSII), Cytochrome_*b*_6_*f*_, and Photosystem I (PSI) to the final electron acceptor NADP^+^ via the mobile electron carriers plastoquinone (PQ), plastocyanin (PC), and ferredoxin (Fd). Thereby a proton gradient is formed, which is used to drive the synthesis of ATP by the ATP synthase. The second part of the photosynthetic process is the Calvin-Benson-Bassham (CBB) cycle, regulated by the thioredoxinsystem (Geigenberger et al., 2017). NADPH and ATP produced by the PETC are used during the CBB cycle to fix CO_2_ into organic compounds. Any imbalance between production and consumption can lead to adverse reactions, such as the production of reactive oxygen species (ROS) (Asada, 2006; Suzuki et al., 2012; Schwarzlander and Finkemeier, 2013) and affect the overall photosynthetic efficiency. Several sub-processes exist, distributed over the whole PETC, that contribute to the production of potentially toxic ROS compounds (Maurino and Flügge, 2008; Dietz et al., 2016; Khorobrykh et al., 2020).

To fine-adjust the formation of ATP and NADPH in the PETC, alternative electron transport pathways evolved (Curien et al., 2016). These alternative electron transport pathways are used to react immediately to changing environmental conditions (Alric and Johnson, 2017). Foremost, the cyclic electron flow (CEF) around PSI including the PGR5-PGRL1 mediated pathway is worth mentioning (Johnson, 2011). Studies have shown that CEF is essential for the functioning of photosynthesis (Munekage et al., 2004) and acts as a protective mechanism in fluctuating light conditions (Kono et al., 2014; Kono and Terashima, 2016). Alternative electron transport pathways balance the ATP and NADPH ratio to prevent an overexcitation of photosystems and redox imbalance in the PETC. Thus, the chance of forming toxic ROS is lowered. The Mehler reaction at PSI, which forms superoxide radicals ${\text{O}}_{2}^{-\cdot }$, was extensively investigated in multiple species (Makino et al., 2002; Curien et al., 2016). Scavenging of ROS, for instance via the ascorbate-glutathione (ASC-GSH) cycle, is potentially an energy-demanding process (Das and Roychoudhury, 2014). However, it prevents physical damage inflicted on the molecular machinery of photosynthesis, which would be even more severe for the energy balance (for an analysis of costs associated with photoinhibition, see for example Raven, 2011). Multiple sophisticated regulatory mechanisms evolved to prevent the formation of ROS beforehand by lowering the energy pressure that acts on the PETC, such as non-photochemical quenching (NPQ) (see Müller et al., 2001).

Because of the existence and possible interaction of numerous mechanisms acting on different parts of the PETC, a system-wide investigation of the dynamics of photosynthesis is necessary. Existing evidence of the beneficial role of various water to water (W-W) cycles during photosynthesis (Curien et al., 2016) inspired us to investigate their impact on balancing the ATP to NADPH ratio. Computational kinetic models of photosynthesis have been proven to be useful for such analyses (Stirbet et al., 2020). Yet, none of these models investigated the role of ROS formation and scavenging. Our goal was to expand the existing model (Matuszyńska et al., 2019) of photosynthesis with key steps of both ROS formation and scavenging (via the ASC-GSH cycle) around PSI as well as linking the W-W cycle with acclimation mechanisms. Moreover, based on our previous supply-demand analyses (Matuszyńska et al., 2019), we have included the regulation of key CBB enzymes through the thioredoxin system. This model thus provides the theoretical background to investigate non-trivial connections of the different components and to study complex systemic behaviour.

In this work we present the results of multiple analyses that allowed us to investigate the importance of alternative electron flows around PSI. We systematically investigated the impact of the Mehler reaction and the CEF on intermediate concentrations of both PETC and CBB cycle. We found out that some of the fluxes in the PETC are drastically influenced by the CEF. Therefore, we performed a Metabolic Control Analysis (MCA) that clearly showed a high impact of the Sedoheptulose-bisphosphate enzyme (SBPase) on the ROS scavenging mechanism, CBB and the PETC. Finally, the role of the SBPase was further elucidated. With this scientific work, we formalised a connection between the CBB cycle, PETC, and ASC-GSH cycle. We showed the interconnection between these parts of photosynthesis and also shed light on the control each part has over others via mathematical modelling. We therefore expanded our understanding of the complex interplay between different acclimatory processes in photosynthesis and created a computational framework to stimulate future scientific efforts in this direction.

## 2. Methods

### 2.1. Model Description

**Figure 1:**
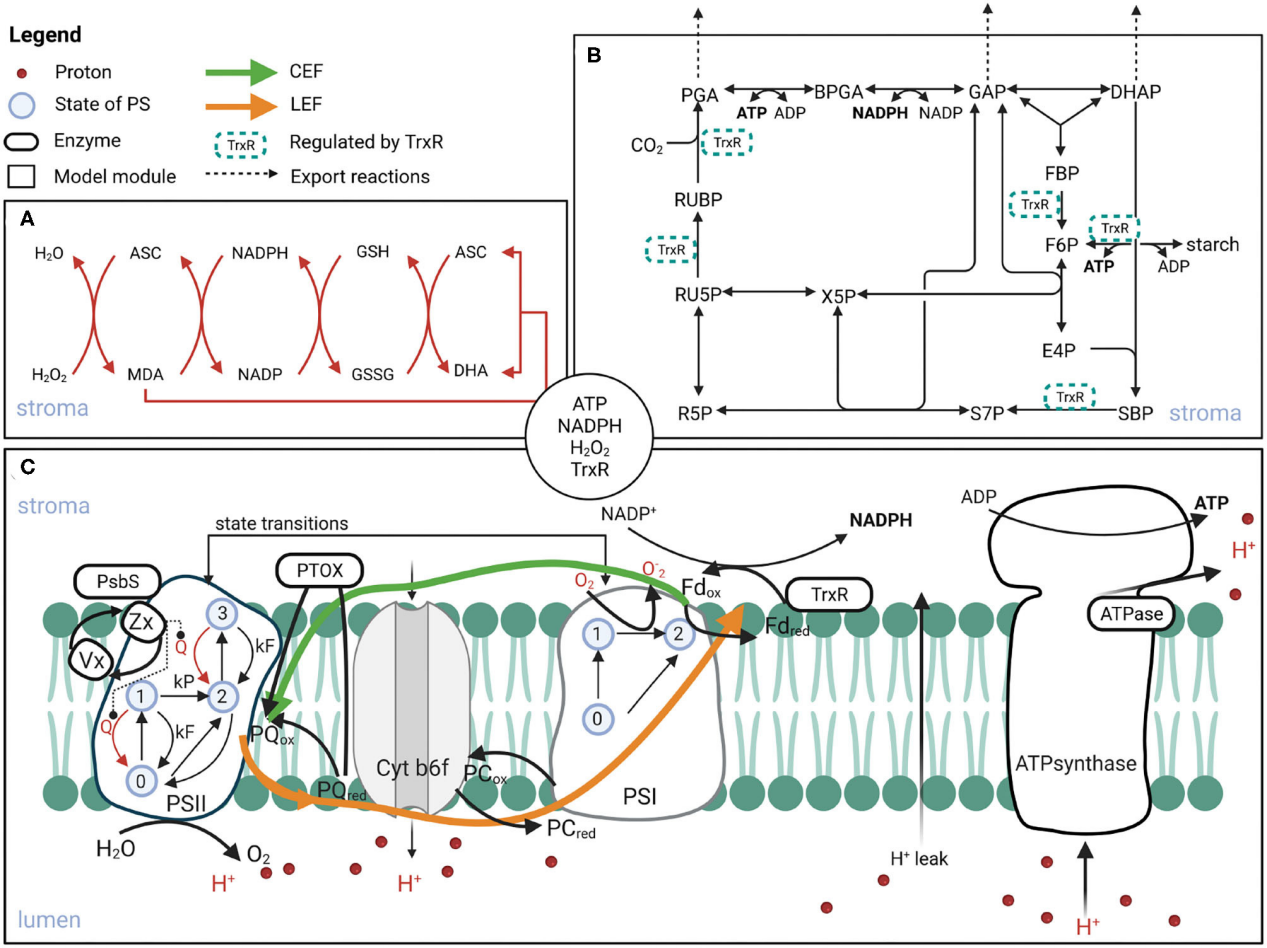
Schematic representation of the processes included in the computational model of photosynthesis. The model consists of three modules: ascorbate-glutathione (ASC-GSH) cycle **(A)**, CBB with TrxR regulated reactions **(B)**, and PETC **(C)**. The compounds in the circle in the centre are the ones exchanged between the compartments. Created with BioRender.com.

We have developed further the previously published mechanistic model of photosynthesis (Matuszyńska et al., 2019). The description of the demand side (Figure 1B) has been firstly complemented by including the thioredoxin reductase (TrxR) regulation. TrxR regulates the activation of the CBB-enzymes, depending on reduced Fd. Next, considering that the CBB cycle is the main, but not the only consumer of the energy equivalents produced by the PETC (Figure 1C), we included two reactions representing additional consumption of ATP and NADPH. Finally, the focus was put on adding two mechanisms responsible for the production and scavenging of ROS around PSI. An alternative electron transfer from PSI to oxygen has been included, leading to the production of superoxide which is rapidly converted to hydrogen peroxide (H_2_O_2_) by the superoxide dismutase (SOD). This implementation required changing the description of the PSI mechanism from the original model (Matuszyńska et al., 2019). Because of the rapid velocity of the SOD enzyme, the H_2_O_2_ production is modelled as a single step, representing the Mehler reaction. We based our simplified description of the ROS scavenging reactions on the published kinetic models of the ASC-GSH cycle by Valero et al. (2009, 2015). Our description of the cycle is represented by four saturating enzymatic reactions [mediated by ascorbate peroxidase (APX), monodehydroascorbate reductase (MDAR), dehydroascorbate reductase (DHAR), glutathione reductase (GR)] and one spontaneous disproportion of monodehydroascorbate radicals (MDA), see Figure 1A. The pools of ASC and GSH are considered constant.

#### 2.1.1. Linear and Alternative Electron Flows

The rates of electron flow through various pathways are directly calculated from the rates through PSII and FNR. In the model, the stoichiometry of the rate of PSII is


(1) $$\begin{array}{r} {\text{H}}_{2}\text{O}+2\text{h}\nu \to 2e^{-}+\frac{1}{2}{\text{O}}_{2}+2{\text{H}}_{\text{lumen}}^{+}, \end{array}$$

which produces 2 electrons. Therefore, the rate of linear electron flow (LEF) is twice the simulated rate through PSII. Likewise, the rate of CEF is twice the rate mediated by FNR.

#### 2.1.2. Units

The choice of units is the same as in Matuszyńska et al. (2019), keeping the original units of stromal and lumenal compartments. The concentrations in the lumen are expressed in mmol (mol Chl)^−1^ and inside the stroma in mM. To convert the concentrations of ATP, NADPH and H_2_O_2_ produced in the lumen to the unit of the stroma, where these metabolites are consumed/scavenged, we employ a conversion factor where 1 mmol (mol Chl)^−1^ corresponds to 3.2·10^−5^ M in the stroma (Laisk et al., 2006).

### 2.2. Computational Analysis

The mathematical model is a system of 30 ordinary differential equations with 46 reaction rates. The model was integrated with Assimulo (Andersson et al., 2015) via the Python-based software modelbase version 1.3.8 (van Aalst et al., 2021). Python files containing the model and Jupyter notebooks with our simulations used to produce all figures are provided on our GitLab repository https://gitlab.com/qtb-hhu/models/cyclicphotosyn-2021.

#### 2.2.1. Metabolic Control Analysis

Flux ($C_{v_{k}}^{J_{j}}$) and concentration ($C_{v_{k}}^{S_{j}}$) control coefficients are defined as


(2) $$\begin{array}{r} C_{v_{k}}^{J_{j}}=\frac{v_{k}}{J_{j}}\frac{\partial J_{j}/\partial p}{\partial v_{k}/\partial p}, \end{array}$$

(3) $$\begin{array}{r} C_{v_{k}}^{S_{j}}=\frac{v_{k}}{S_{j}}\frac{\partial S_{j}/\partial p}{\partial v_{k}/\partial p}, \end{array}$$

where *J*_*j*_ and *S*_*j*_ are respectively the steady-state fluxes and concentrations of the system, *p* is a kinetic parameter which affects directly only reaction *k* with the rate *v*_*k*_ (see Kacser and Burns, 1973; Heinrich and Rapoport, 1974; Heinrich and Schuster, 1996). We approximated these formulas numerically using the central difference, varying the parameters by ±1%. Control coefficients quantify the relative effect of a parameter perturbation on steady state fluxes and concentrations.

## 3. Results

**Figure 2:**
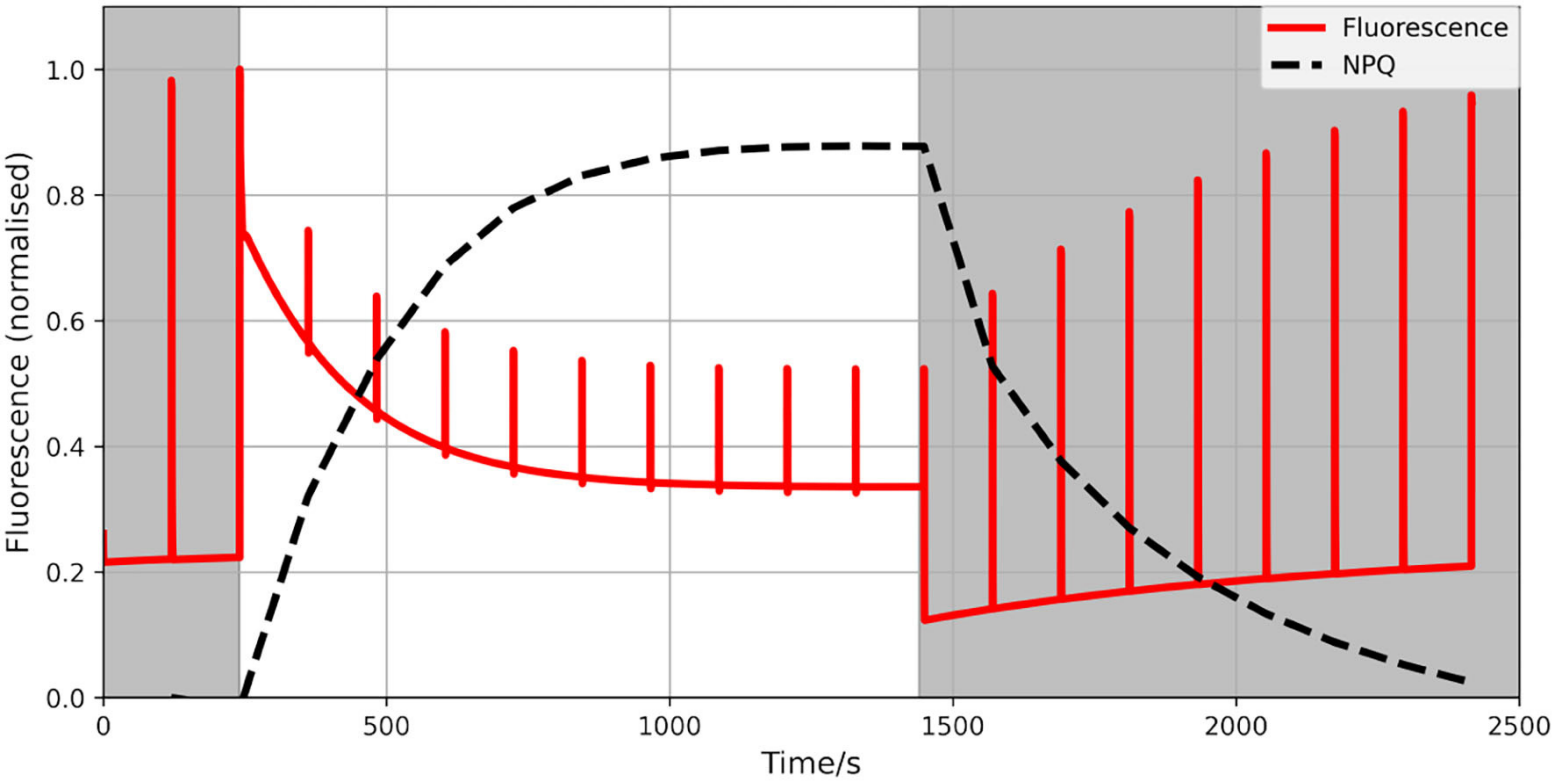
Calculated fluorescence trace (red) of a PAM experiment of a generic photosynthetic organism without CEF. A standard dark-light-dark protocol was simulated (dark phase shaded). We used 1,000 μmol m−2s−1photosynthetic photon flux density (PPFD) as the intensity of the actinic light and 40 μmol m−2s−1PPFD for dark/dimmed light with pulses every 2 min. The calculated NPQ is marked in black (dashed). It exhibits well-known dynamics of excitation and relaxation.

The model has been used to study electron flows around PSI and their relevance to the overall performance of the photosynthetic machinery under both steady-state and dynamic conditions. To confirm that our improved model can indeed be used beyond steady-state and can realistically reproduce short-term acclimation responses we simulated a standard PAM fluorescence trace. The results exhibit typical fluorescence dynamics under high light conditions (Figure 2). It should be however noted that quantities discussed here should not be understood as precise predictions of a specific experimental observations, but are rather meant to illustrate the general plausibility of the model behaviour.

### 3.1. Steady-State Behaviour Under Continuous Light

**Figure 3:**
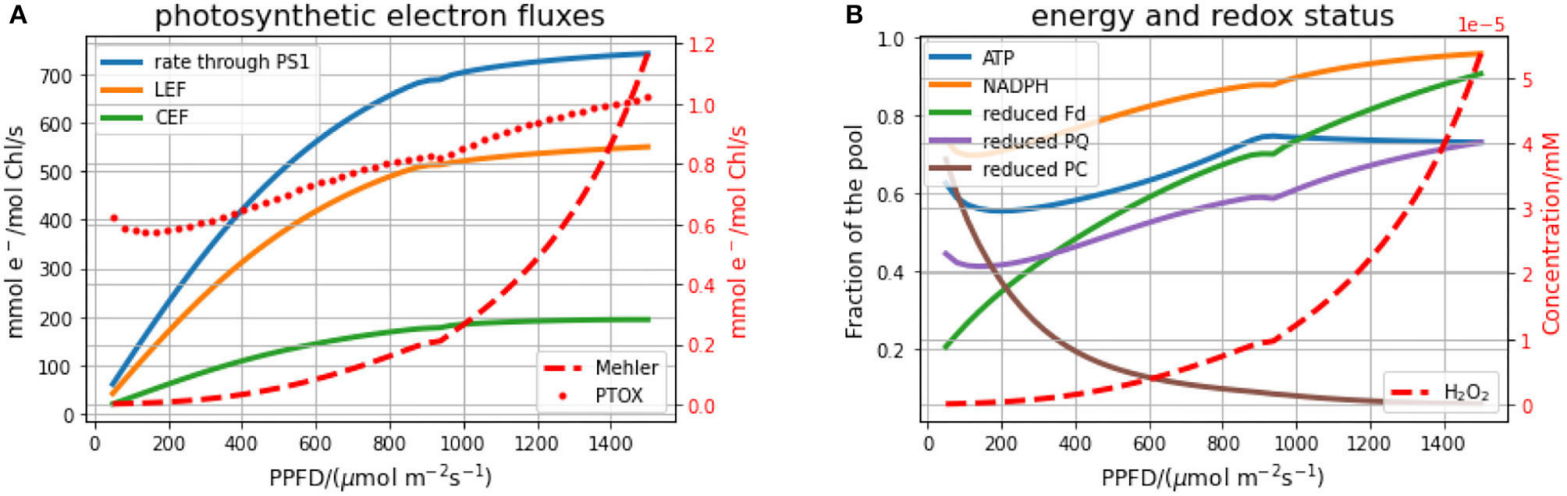
Stationary fluxes and stationary energy and redox status of the electron transport chain for different intensities of constant light. **(A)** displays the electron flux through PSI, linear (LEF) and cyclic electron flow (CEF), the Mehler reaction and the plastid terminal oxidase (PTOX). **(B)** displays the energy equivalents (ATP, NADPH) and redox states of the electron chain (PQ, PC, Fd), as well as the stationary H_2_O_2_ concentration resulting from the Mehler reaction.

We first investigate the steady-state behaviour of the model under various light intensities (Figure 3). In the left panel (Figure 3A), the stationary electron fluxes over different light intensities through the PSI, LEF, CEF, the Mehler reaction and the plastid terminal oxidase (PTOX) are depicted. The rate of the electron transport chain increases linearly for low light conditions and saturates in high light. Carbon fixation rates follow the same general pattern (see Supplementary Material), which has been repeatedly confirmed in experiments for a wide range of photosynthetic organisms (Hesketh and Baker, 1967; Huang et al., 2016). In our simulations, the transition to the light-saturated regime occurs around a photosynthetic photon flux density (PPFD) of 900 μmol m−2s−1, which is in good agreement with previously observed and modelled values (Kromdijk et al., 2019). In contrast to the electron transport chain, the rate of the Mehler reaction strongly increases in high light conditions, leading also to increased stationary hydrogen peroxide (H_2_O_2_) concentrations (Figure 3B). Nevertheless, even in high light, the rate of the electron transfer to oxygen by the Mehler reaction reaches only around 0.2% of the electrons transferred by PSI. This means that even under high light, less than 1% of the NADPH produced by the electron transport chain is required to scavenge the ROS produced in PSI through the Mehler reaction. Most redox carriers are more reduced in high light, with the exception of PC, which is more oxidised in higher light. This observation can be explained by the fact that more light increases the rate of PSI, which directly removes electrons from the PC pool. This explanation is supported by the results of the MCA, indicating that increased PSI leads to a more oxidised PC pool (see also **Figure 6**).

A key enzyme in the ASC-GSH cycle is the MDA reductase, which reduces MDA back to ASC using NADPH as an electron donor (Figure 1A). Interestingly, a simulated knock-down of this enzyme to 1% of its original value does not affect the overall electron fluxes. However, in high light, the deficiency in MDA reductase is compensated by the spontaneous disproportionation of MDA into DHA and ASC, which leads to approximately 100-fold increased levels of the MDA radical (see Supplementary Material). Because overall electron fluxes and H_2_O_2_ production rates are not affected, also the ratio of NADPH required for scavenging ROS is unaltered in the MDA reductase knock-down.

### 3.2. Performance of PGR5 Mutants Under Continuous Light

**Figure 4:**
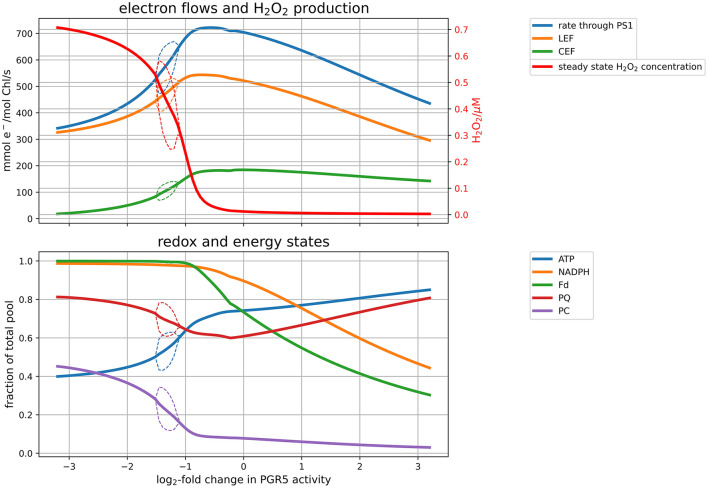
Response of the model under high light (PPFD 1,000 μmol m^−2^s^−1^) to changes in CEF activity. Altered CEF activity was simulated by changing the rate constant for PGR5, the enzyme transferring electrons from Fd to PQ. The top panel displays electron flows through the PETC and the H_2_O_2_ concentrations resulting from the flux through the Mehler reaction and the ASC-GSH cycle. The bottom panel shows the energy (ATP) and redox state (NADPH, Fd, PQ, PC) of the system. In both panels, the solid lines indicate stationary values. The thin dashed lines indicate a parameter range, in which limit cycle oscillations were observed, denoting the minimum and maximum values of the oscillating variable. Outside these parameter regions, the solid line indicates stationary values, within the bubble averages over oscillations.

By transferring electrons from Fd back into the PQ pool, the protein PGR5 mainly mediates the CEF. We employed our model to study how altering the CEF affects electron flows and downstream metabolism, by systematically varying the corresponding enzyme activity (Figure 4) under simulated high light conditions (PPFD 1000 μmol m−2s−1). These simulations correspond to knocking down (KD) or overexpressing (OE) the PGR5 protein, which catalyses the reduction of PQ by Fd. Slowing down CEF does not only result in a slower CEF rate but also leads to a reduced overall photosynthetic electron flux and carbon fixation rate (top panel of Figure 4). This behaviour illustrates the physiological role of CEF to adjust the ATP/NADPH ratio produced by the PETC to the downstream demand. Because the provided ratio does not align with downstream demand, electrons accumulate in the final products of the PETC, leading to over-reduced Fd and NADPH pools (lower panel of Figure 4). Over-reduced Fd, in turn, reduces the availability of electron acceptors for PSI, which leads to an increased rate of the Mehler reaction and H_2_O_2_ levels. The reduced photosynthetic capacity of PGR5 mutant plants has been demonstrated experimentally (DalCorso et al., 2008). A simulated knockout (KO) quantitatively reproduces the observation that maximal PSII rate is approximately half of the wildtype (~ 300 vs. 520 mmol e^−^/mol Chl/s in Figure 4), and that light saturation is reached at lower intensities compared to the wildtype (approximately at PPFD 500 μmol m−2s−1—see Supplementary Material). Also increasing the CEF has negative effects on the performance. If more electrons are re-inserted into the PETC, the overall ATP level increases and electron carriers are less reduced, but the overall production rate of NADPH and ATP decreases, leading again to a reduced carbon fixation rate. It seems, therefore, that there exists an optimal PGR5 activity, that maximises photosynthetic efficiency and carbon fixation by avoiding over-reduction of the electron chain, while at the same time redirecting not more electrons than necessary back into the chain. Under low light (for figures, see Supplementary Material), the CEF plays a less important role. Under these conditions, increasing PGR5 activity increases the ratio of CEF to LEF and slightly decreases carbon fixation rates. The simulations suggest that, whereas under high light CEF is clearly beneficial for the photosynthetic efficiency, under low light conditions a low PGR5 activity is favourable for CO_2_ fixation rates.

### 3.3. Importance of Alternative Electron Flows Under Fluctuating Light

**Figure 5:**
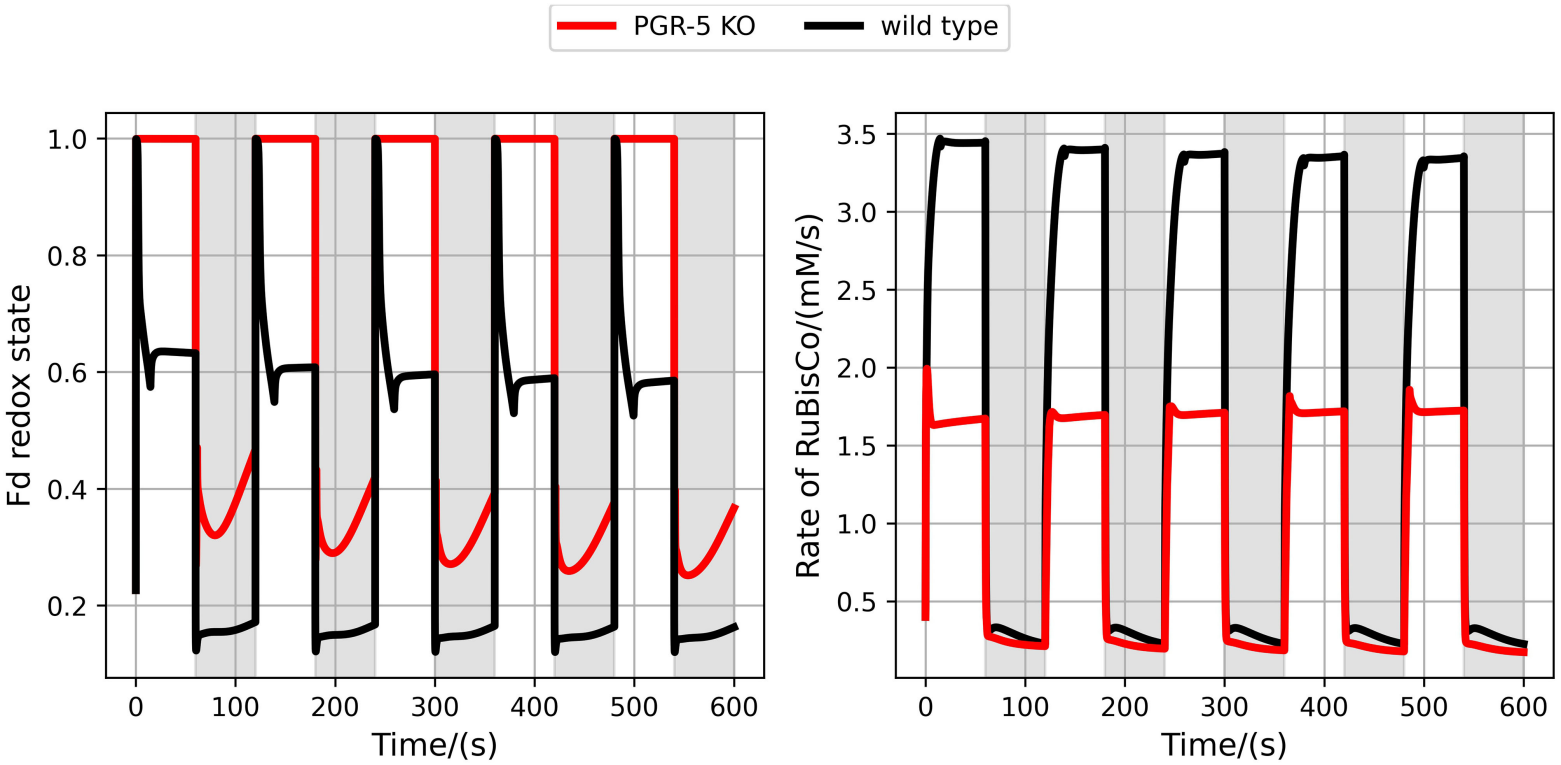
Simulating fluctuating light for the wildtype and the PGR5 knockout (KO) mutant. Shown are the Fd redox state **(left)** and the RuBisCO activity **(right)** for the wildtype (black) and the PGR5 knockout (red). Light intensities were changed every 60s between high light (PPFD 600 μmol m−2s−1, white regions) and low light (PPFD 40 μmol m−2s−1, grey regions).

It was repeatedly demonstrated experimentally that the CEF is particularly important to maintain photosynthetic activity under fluctuating light conditions (Yamori et al., 2016; Yamamoto and Shikanai, 2018). Comparing simulations of wildtype with PGR5 mutant shows that carbon fixation is indeed drastically reduced when no CEF operates (Figure 5). These results are in qualitative agreement with experimental findings (Yamori et al., 2016). However, the experimentally observed dynamics are quantitatively different from our simulations. In particular, the reactivation dynamics of RuBisCO in the transition to high light are considerably slower in the experiment as compared to the model simulations. This indicates that the mechanisms activating the CBB cycle in such transitions are not yet represented in the model in a quantitatively correct way. Still, the model provides a theoretical explanation for the reduced photosynthetic efficiency by illustrating that the PGR5 mutant is unable to establish a healthy redox balance in light periods.

### 3.4. SBPase Exhibits Striking Control Over Photosynthesis Under High Light

**Figure 6:**
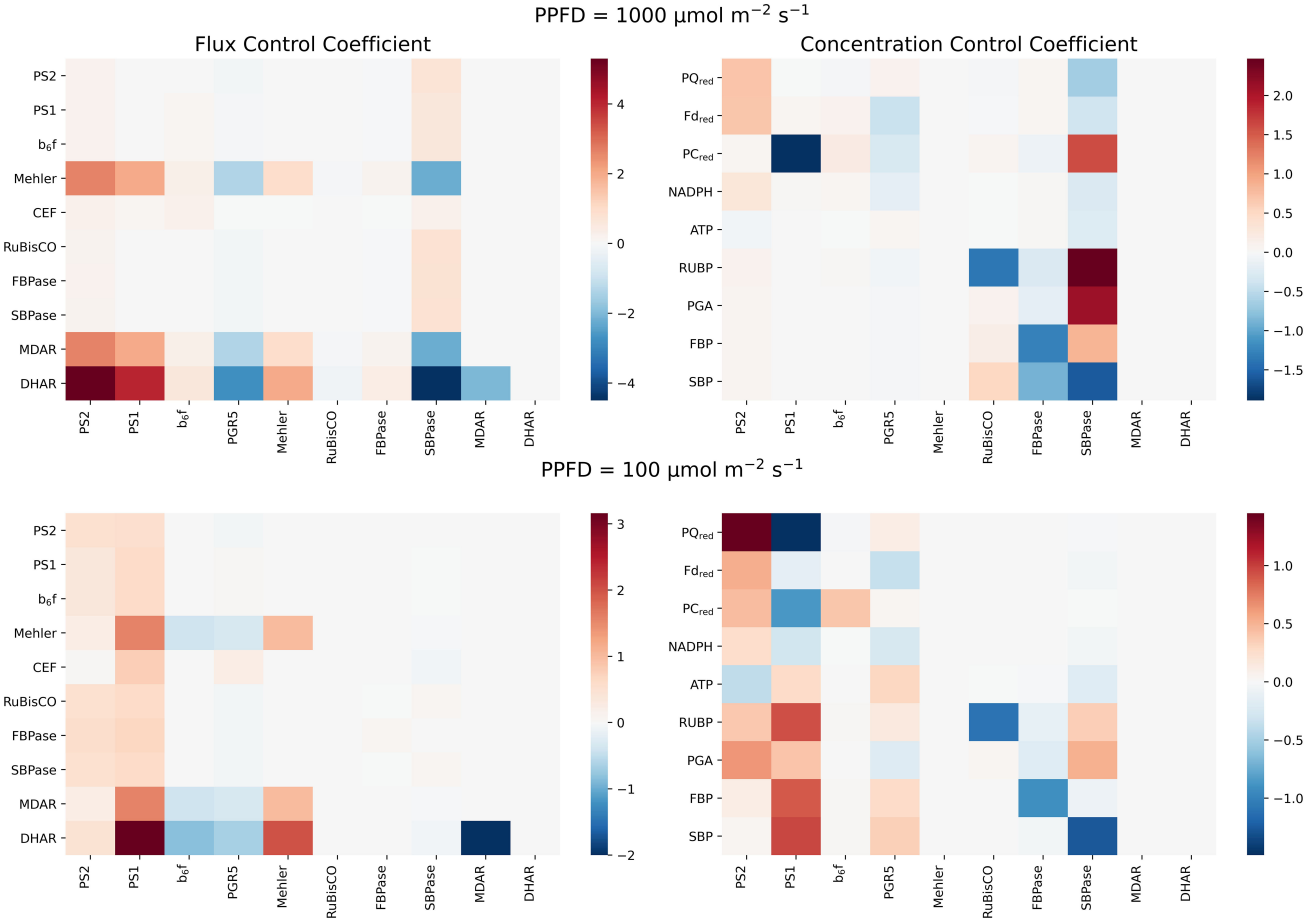
Results of the MCA. Flux **(left)** and concentration **(right)** control coefficients of representative reactions of photosynthesis in high and low light conditions. The top panels show the distribution of control under high light (PPFD 1,000 μmol m−2s−1), the bottom panels under low light (PPFD 100 μmol m−2s−1). For all panels, on the x-axis we marked the perturbed parameters. The parameters are perturbed by ± 1% and the resulting effect on steady state values is monitored for the quantities on the y-axis. It can be clearly seen how the control of photosynthesis shifts from the supply side in low light **(bottom)** to the demand side under high light, exhibiting striking control of SBPase **(top)**.

The above investigations illustrate that electron flow around PSI apparently affects not only the PETC itself but also downstream metabolism, in particular carbon fixation. In order to understand which processes carry the strongest control in this complex supply-demand system, we performed MCA and systematically determined flux and concentration control coefficients for high (PPFD 1000 μmol m^−2^s^−2^) and low (PPFD 100 μmol m^−2^s^−2^) light conditions. A selection of flux and concentration control coefficients are depicted in Figure 6. Additionally to get a global picture of the model's behaviour we performed a simple golbal sensitivity analysis using Latin Hyperspace Sampling and Partial Rank Correlation Coefficients that can be found in the Supplementary Material.

In agreement with the analysis of the effects of perturbing PGR5 activity, and thus CEF (Figure 4), it is observed that increasing PGR5 leads to slightly decreased fluxes in the PETC and the CBB cycle. In contrast, increasing CEF strongly decreases the Mehler reaction and the associated scavenging pathways. Remarkably, under high light, the strongest control on PETC and CBB cycle fluxes is exhibited by the SBPase, whereas RuBisCO carries almost no flux control. This observation confirms previous theoretical results obtained from a model simulating the CBB cycle alone (Poolman et al., 2000). Increasing SBPase results in a significant increase of both PETC and CBB cycle rates, and strongly suppresses the Mehler reaction and associated scavenging reactions, while the redox pools except PC are more oxidised, and ATP levels are decreased. PSII is the initial complex of the PETC and thus a natural candidate for high flux control. Indeed, it exerts positive control over PETC and CBB cycle fluxes in high light, but with a much lower control strength compared to SBPase. Increasing PSII (and PSI and to a lesser extent the cytochrome b_6_f complex) predominantly increases the Mehler reaction. This behaviour changes dramatically under low light. Here, CBB enzymes exert almost no flux control, but electron transport and carbon fixation rates are mostly controlled by the activities of the photosystems. Increasing PSII leads to more reduced redox pools and lower ATP levels, whereas increasing PSI leads to more oxidised redox pools and higher ATP levels. Both photosystems have a positive control on CBB cycle intermediates RuBP and PGA, while only PSI positively affects the bisphosphates FBP and SBP. Altogether these analyses confirm the previous observation (Matuszyńska et al., 2019) that under low light control resides predominantly on the supply side (PETC), while under high light control is shifted toward the demand side (CBB).

### 3.5. ROS Production as a Balancing Mechanism

**Figure 7:**
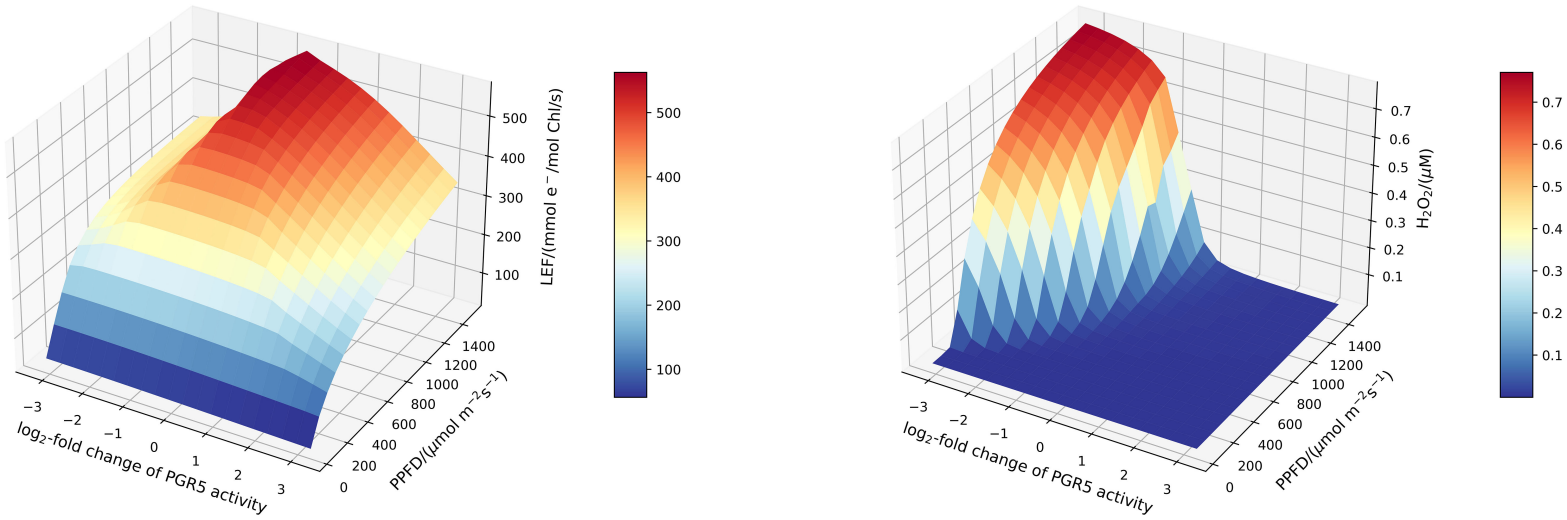
Surface plots of the stationary linear electron flux **(left)** and H_2_O_2_ concentration **(right)** in response to altered PGR5 (CEF) activity and light intensity. Increased CEF activity reduces stationary H_2_O_2_ concentrations. For higher light intensities, more CEF activity is required to maintain low H_2_O_2_ levels. This indicates that CEF activity should be regulated for maximal efficiency under various light regimes.

To increase our understanding of the antagonistic behaviour of the Mehler reaction and the CEF, and to account for the changing relative importance of these processes under low and high light, we systematically investigated the efficiency of photosynthesis for altered CEF under different light intensities. Figure 7 displays simulated linear electron fluxes and H_2_O_2_ concentrations in response to changed light intensities and PGR5 activities. Whereas under low light conditions (of less than approximately 500 μmol m−2s−1, the photosynthetic efficiency is rather independent of the PGR5 activity, this is dramatically different in high light. Both, too low and too high CEF activity leads to a reduced photosynthetic flux, but for different reasons. Impaired CEF results in drastically elevated H_2_O_2_ levels because ATP and NADPH production ratios cannot be adapted to the downstream requirements. In contrast, increased activity of PGR5 mediated CEF simply leads to more oxidised NADPH and Fd (see Figure 5), and redirects electron flux from linear to cyclic, thus reducing the overall net carbon fixation rate.

## 4. Discussion

In oxygenic photosynthesis, LEF is considered the basic driver of photosynthetic carbon fixation. Yet alone, it does not provide the exact ratio of ATP to NADPH that is necessary to drive carbon assimilation (Kramer and Evans, 2009). Hence, alternative circuits of the electron flow are considered to balance the production of ATP per NADPH (Curien et al., 2016). In this work the presented computational model has been developed to investigate the alternative electron circuits around PSI that produce a proton gradient without NADPH synthesis, therefore altering this ratio. These include the CEF around PSI and two of the W-W cycle including the Mehler reaction at PSI and the PTOX downstream PSII (Curien et al., 2016). Additionally, we have provided an important link between ROS formation and metabolism regulation by including a simple description of ROS scavenging around PSI via the ASC-GSH cycle. This allowed us to further investigate the role of the cycle in keeping photosynthetic activity at medium and higher light intensities (Muller-Schussele et al., 2020). Although it is only one of the many known pathways (Maurino and Flügge, 2008), it is considered as the first step in the long process of including redox balance through ROS production into computational models of photosynthesis, in an attempt to support the synthetic redesign of photosynthetic systems (Zhu et al., 2020).

We have argued before that photosynthesis shall be viewed as a supply-demand system because of the connection between the ATP and NADPH production and consumption (Matuszyńska et al., 2019). Considering the tight regulation of such a system, we investigated the influence of alternative electron pathways on the rate of CO_2_ assimilation, with a particular focus on their photoprotective behaviour and the role of the CEF (see change in rate of RuBisCO in Figure 5). The presented model is intended to serve as a theoretical workbench that is not only valid for a single experiment or plant species but is in principle adaptable to a wide range of scenarios and photosynthetic organisms. While not precisely calibrated to a particular experimental dataset, we ensured that the model displays realistic behaviour. In particular, the steady-state of key variables, such as the redox state of electron carriers as well as carbon fixation fluxes are plausible, and the simulated PAM experiments show characteristic NPQ dynamics (Figure 2). The model allows moreover the simulation of genetic perturbations, such as KO, KD and OE, which has been demonstrated extensively on the PGR5 mutant, impaired with its capacity of a CEF. The focus on the PGR5/PGRL1 pathway was motivated by its particular role in regulating proton motive force around PSI (Wang et al., 2015). Figure 4 highlights the critical role of the CEF by displaying a strongly reduced LEF, highly oxidised redox state of the electron carriers and a very strong increase in hydrogen peroxide concentration. Interestingly, our computational analysis systematically displayed the dependency of the system behaviour in PGR5 KO and OE to different light intensities. The differences between PGR5 mutants are mostly visible in higher light conditions, as shown in Figure 7.

Light, although necessary to drive photosynthesis, can be also harmful to the organism. NPQ is a central part of the first line of defence of plants against damaging effect of light. In order to prevent high ROS levels, plants developed mechanisms allowing dissipation of excess light energy as heat (Ruban, 2016). Our simulations demonstrate that in high light intensity the whilst Mehler and PTOX reactions continue to increase, contributing significantly to the photoprotection and overall redox balance (Figure 3). These results are in line with the previously proposed role of the W-W cycle acting as a relaxation system to suppress the photoproduction of ^1^O_2_ in PSII (Asada, 2006). We expect that the model presented in this work will be useful for a systematic assessment of the possible beneficial effect of ROS formation in a physiological context (Foyer and Noctor, 2005; Foyer, 2018; Mhamdi and Van Breusegem, 2018).

Within our expanded model of photosynthesis we have performed MCA and confirmed the pivotal role of SBPase in control over the system, as in our previous work (Poolman et al., 2000; Matuszyńska et al., 2019). SBPase has been shown to control both supply and demand of photosynthesis and, consequently, in this expanded model, it exhibits a strong influence on the electron flows. Figure 6 displays that in high light conditions, an increase of SBPase activity strongly decreases the Mehler reaction rate and as a consequence the rate of the main scavenging reactions DHAR and MDAR. This phenomenon can be explained by the increase in efficiency of the CBB cycle, which causes faster ATP consumption and prevents over-reduction of the PETC, therefore reducing the rate and impact of the Mehler reaction. It is important to consider that this behaviour is observed in scenarios with saturated carbon dioxide conditions. However, the model can in principle be directly applied to other, more natural, conditions. For example, it would be interesting to compare the electron flux distribution under non-saturating conditions. Further, although we have varied oxygen systematically, to mimic conditions under which oxygen becomes limiting, all our analysis have been performed under saturated CO_2_ conditions.

A natural further step of expanding this work would be to include the mechanism of photorespiration, mainly because it plays a physiological role in reducing the redox pressure in the stroma under conditions leading to low carbon fixation (Ort and Baker, 2002) and because it is a major source of ROS associated with the photosynthetic activity (Dietz et al., 2016). A reliable mathematical model of photorespiration to be considered has been proposed by Yokota et al. (1985).

With this work we provide a tool to further study the dynamics and cross-talk between the multiple regulatory mechanisms activated by photosynthetic organisms in response to changes in light. With our model, we could demonstrate how electron flows around PS1 affect photosynthetic efficiency and how increasing CBB cycle activity decreases Mehler reaction activity. Moreover, the model allowed us to rationalise that CEF should be regulated with changing light intensities as a trade-off between optimising electron flux efficiency and minimising ROS production. We envisage that this model helps to further investigate the tight relation between ROS scavenging in the chloroplast and the dynamic adaptation of photosynthesis to changing conditions.

## Data Availability Statement

The original contributions presented in the study are included in the article/Supplementary Material, further inquiries can be directed to the corresponding author/s.

## Author Contributions

AM: initial idea and conceptualisation. AM and OE: funding acquisition. AM, MA, and NS: visualisation. AM, OE, MA, NS, and TN: model building. AM, BH, OE, MA, NS, and TN: formal analyses. BD and TN: writing—original draft and introduction. BH: writing—original draft and methods. OE and NS: writing—original draft and results. AM and NS: writing—original draft, discussion, and writing—review and editing. All authors read and accepted the final version of the manuscript.

## Funding

This work was funded by the Deutsche Forschungsgemeinschaft (DFG, German Research Foundation) under Germany's Excellence Strategy - EXC-2048/1 - project ID 390686111 (AM and OE), the Deutsche Forschungsgemeinschaft Research Grant MA 8103/1-1 (AM), the Deutsche Forschungsgemeinschaft (DFG), project ID 391465903/GRK 2466 (TN), and the EU's Horizon 2020 research and innovation programme under the Grant Agreement 862087 (MA).

## Conflict of Interest

The authors declare that the research was conducted in the absence of any commercial or financial relationships that could be construed as a potential conflict of interest.

## Publisher's Note

All claims expressed in this article are solely those of the authors and do not necessarily represent those of their affiliated organizations, or those of the publisher, the editors and the reviewers. Any product that may be evaluated in this article, or claim that may be made by its manufacturer, is not guaranteed or endorsed by the publisher.

## Abbreviations

ASC: ascorbate
CBB: Calvin-Benson-Bassham
CEF: cyclic electron flow
OE: overexpressor
Fd: ferredoxin
GSH: glutathione
LEF: linear electron flow
KD: knock down
KO: knock out
MCA: Metabolic Control Analysis
MDA: monodehydroascorbate radicals
PETC: photosynthetic electron transport chain
PC: plastocyanin
PPFD: photosynthetic photon flux density
PSI: photosystem I
PSII: photosystem II
PTOX: Plastid terminal oxidase
PQ: plastoquinone
ROS: reactive oxygen species
SOD: superoxide dismutase
TrxR: thioredoxin reductase
W-W: water-water.
