# Supplementary material for: Computational Analysis of Alternative Photosynthetic Electron Flows Linked With Oxidative Stress
Source: Front Plant Sci. 2021 Oct 22;12:750580. doi: 10.3389/fpls.2021.750580 (PMC8569387; doi:10.3389/fpls.2021.750580)
Supplement: Supplementary file 1 [file Data_Sheet_1.pdf]

# Supplementary Material:
# Computational analysis of alternative photosynthetic electron flows linked with oxidative stress

Supplementary information to the manuscript by Saadat *et al.* ”Computational analysis of alternative photosynthetic electron flows linked with oxidative stress”.

## 1 MODEL CONSTRUCTION

Presented computational model of photosynthesis has been developed using `modelbase` package (van Aalst et al., 2021) and has been assembled in a modular way. The core of the model consists of the reactions of the generic model of C3 photosynthesis published by Matuszyńska et al. (2019) and described in file matuszynska.py (`https://gitlab.com/qtb-hhu/models/cyclicphotosyn-2021/-/tree/master/models`). All kinetic parameters from Matuszyńska et al. (2019) have remained unchanged. By adding modules *mehler* (describes the dynamics of the ROS formation via the Mehler reaction and ROS scavenging via the ascorbate-glutathione cycle), *thioredoxin* (adds thioredoxin regulation of key enzymatic reactions of the Calvin-Benson-Bassham cycle), and *consumption* (describes the additional cell demand of ATP and NADPH), a full model analysed in this work is assembled. New parameters are provided in tables below (Tab. S1-S2).

### 1.1 mehler: ROS formation and scavenging

An alternative electron transfer from PC to oxygen has been included, leading to the production of superoxide which is rapidly converted to hydrogen peroxide by the superoxide dismutase. This implementation of ROS formation via Mehler reaction required changing the description of the PSI from the original model Matuszyńska et al. (2019), according to the scheme Figure S1.

The simplified description of the ROS scavenging reactions was based on the published kinetic models of the ascorbate-glutathione cycle (Valero et al., 2009, 2015). Implemented steps are depicted in Figure S2 and kinetic values used in this work are provided in Table S1.

### 1.2 thioredoxin: Thioredoxin regulation

The description of the demand side of the model, the Calvin-Benson-Bassham (CBB) cycle, has been expanded by including the thioredoxin reductase (TrxR) regulation. TrxR regulates the activation of the CBB-enzymes, depending on oxidized Fd and kinetic values used in this work are provided in Table S2.

### 1.3 consumption: Additional ATP and NADPH demand

Considering that the CBB cycle is the main, but not the only consumer of the energy equivalents produced by the photosynthetic electron transport chain, we included two simple mass action reactions representing additional consumption of ATP and NADPH. The kinetic values used in this work are provided in Table S3.

## 2 SUPPLEMENTARY ANALYSES

Additional results to the manuscript, referred through the text as Supplement Material have been collected in a form of two Jupyter Notebooks (supplementary.ipynb and supplementary-gsa.ipynb) and placed in the GitLab repository: `https://gitlab.com/qtb-hhu/models/cyclicphotosyn-2021/-/tree/master/analyses-paper`.

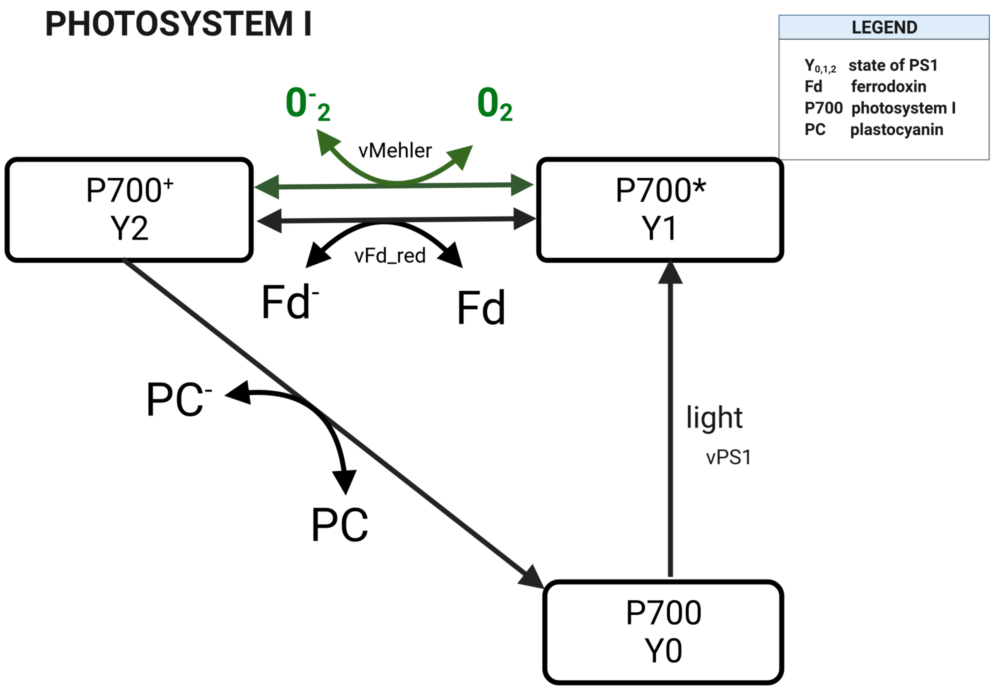


**Figure S1.** Schematic representation of the reactions within Photosystem I. $Y_0$ (P700) is activated by light to $Y_1$ (P700*), which reduces ferredoxin (Fd) or produces oxygen (O2, via the Mehler) with its concomitant oxidation to $Y_2$ ($P700^+$). The oxidised state is reduced back to its ground state by plastocyanin (PC).

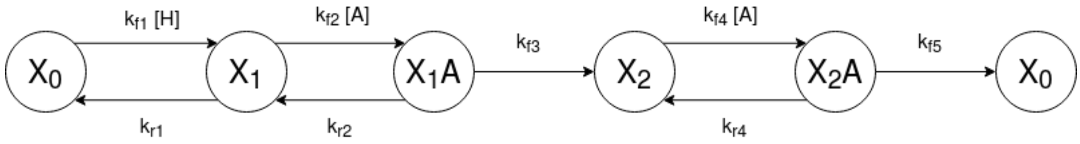


**Figure S2.** Schematic representation of the ascorbate-glutathione cycle based on the computational models by Valero et al. (2009, 2015)

## REFERENCES


Matuszyńska, A., Saadat, N. P., and Ebenhöh, O. (2019). Balancing energy supply during photosynthesis–a theoretical perspective. *Physiologia plantarum* 166, 392–402. doi:10.1111/ppl.12962

Valero, E., González-Sánchez, M. I., Maciá, H., and García-Carmona, F. (2009). Computer simulation of the dynamic behavior of the glutathione-ascorbate redox cycle in chloroplasts. *Plant physiology* 149, 1958–1969. doi:10.1104/pp.108.133223

Valero, E., Macià, H., De la Fuente, I. M., Hernández, J.-A., González-Sánchez, M.-I., and García-Carmona, F. (2015). Modeling the ascorbate-glutathione cycle in chloroplasts under light/dark conditions. *BMC Systems Biology* 10, 11. doi:10.1186/s12918-015-0239-y

van Aalst, M., Ebenhöh, O., and Matuszyńska, A. (2021). Constructing and analysing dynamic models with modelbase v1. 2.3: a software update. *BMC bioinformatics* 22, 1–15. doi:10.1186/s12859-021-04122-7

**Table S1.** Rate constants and key parameters of the ascorbate-glutathione redox cycle

| Parameter | Value | Description and reference |
|---|---|---|
| $kf1$ | 10000.0 | Estimated |
| $kr1$ | 220.0 | BRENDA database |
| $kf2$ | 10000.0 | Estimated |
| $kr2$ | 4000.0 | BRENDA database |
| $kf3$ | 2510.0 | BRENDA database |
| $kf4$ | 10000.0 | Estimated |
| $kr4$ | 4000.0 | BRENDA database |
| $kf5$ | 2510.0 | BRENDA database |
| $XT$ | 0.07 | Concentration of ascorbate peroxidase. Valero et al. (2009) |
| $k_{\mathrm{Mehler}}$ | $1\ (\mathrm{mM})^{-1}\ \mathrm{s}^{-1}$ | Rate constant for summarized hydrogen peroxide production. Estimated. |
| $k_{\mathrm{cat}_{\mathrm{GR}}}$ | $595\ \mathrm{s}^{-1}$ | Turnover rate of gluthation reductase. Valero et al. (2009) |
| $k_{\mathrm{cat}_{\mathrm{DHAR}}}$ | $142\ \mathrm{s}^{-1}$ | Turnover rate of dehydroascorbate reductase. Valero et al. (2009) |
| $k3$ | $500\ (\mathrm{mM})^{-1}\ \mathrm{s}^{-1}$ | Rate constant for the spontaneous disproportion of MDA Valero et al. (2009) |
| $K_{\mathrm{m}_{\mathrm{NADPH}}}$ | 3e-3 mM | Michaelis Menten constant of NADPH. Valero et al. (2009) |
| $K_{\mathrm{m}_{\mathrm{GSSG}}}$ | 0.2 mM | Michaelis Menten constant of oxidized gluthation. Valero et al. (2009) |
| $K_{\mathrm{m}_{\mathrm{D}}\mathrm{HA}}$ | 70e-3 mM | Michaelis Menten constant of dehydroascorbate. Valero et al. (2009) |
| $K_{\mathrm{m}_{\mathrm{GSH}}}$ | 2.5 mM | Michaelis Menten constant of reduced gluthation. Valero et al. (2009) |
| $K$ | $0.5\ (\mathrm{mM})^2$ | Dissociation constant of dehydroascorbate reductase. Valero et al. (2009) |
| $\mathrm{Glutathion}_{\mathrm{total}}$ | 10 mM | Total concentration of reduced and oxidized glutathione. |
| $\mathrm{Ascorbate}_{\mathrm{total}}$ | 10 mM | Total concentration of reduced and oxidized ascorbate. |
| $k_{\mathrm{cat}_{\mathrm{MDAR}}}$ | $300.0\ \mathrm{s}^{-1}$ | Turnover rate of monodehydroascorbate reductase. Valero et al. (2015) |
| $K_{\mathrm{m}_{\mathrm{MDAR-NADPH}}}$ | 23e-3 mM | Michaelis-menten constant of monodehydroascorbate for the conversion to NADPH. Valero et al. (2015) |
| $K_{\mathrm{m}_{\mathrm{MDAR-MDA}}}$ | 1.4e-3 mM | Michaelis-menten constant of monodehydroascorbate for the conversion to MDA. Valero et al. (2015) |
| $\mathrm{GR}_0$ | 1.4e-3 mM | Concentration of gluthatione reductase. Valero et al. (2009) |
| $\mathrm{DHAR}_0$ | 1.7e-3 mM | Concentration of dehydroascorbate reductase. Valero et al. (2009) |
| $\mathrm{MDAR}_0$ | 2e-3 mM | Concentration of monodehydroascorbate reductase. Valero et al. (2015) |

**Table S2.** Rate constants and key parameters of the thioredoxin reductase.

| Parameter | Value | Description and reference |
|---|---|---|
| $\mathrm{thioredoxin}_{\mathrm{tot}}$ | 1 | Relative total concentration of thioredoxin. |
| $e_{\mathrm{cbb}_{\mathrm{tot}}}$ | 6 mM | Maximal concentration of CBB enzymes. Estimated |
| $k_{\mathrm{fd}_{\mathrm{tr}_{\mathrm{reductase}}}}$ | $1\ \mathrm{s}^{-1}$ | Rate constant of ferredioxin thioredoxin reductase. Estimated |
| $k_{e_{\mathrm{cbb}_{\mathrm{activation}}}}$ | 1 | Rate constant of CBB activation. Estimated |
| $k_{e_{\mathrm{cbb}_{\mathrm{relaxation}}}}$ | $0.1\ \mathrm{s}^{-1}$ | Rate constant of CBB relaxation. Estimated |

**Table S3.** Rate constants and key parameters of the NADPH/ATP consumption.

| Parameter | Value | Description and reference |
|---|---|---|
| $k_{\mathrm{ex}_{\mathrm{atp}}}$ | $0.2\ \mathrm{s}^{-1}$ | General consumption rate of ATP. Estimated |
| $k_{\mathrm{ex}_{\mathrm{nadph}}}$ | $0.2\ \mathrm{s}^{-1}$ | General consumption rate of NADPH. Estimated |
